# Unraveling the Neuropsychological Underpinnings of Self-Regulation Problems in Individuals Convicted of Sexual Offenses Against Children: A Look Into Reinforcement Learning

**DOI:** 10.5964/sotrap.7503

**Published:** 2023-05-05

**Authors:** Tineke Dillien, Inti A. Brazil, Bernard Sabbe, Kris Goethals

**Affiliations:** 1 Collaborative Antwerp Psychiatric Research Institute (CAPRI), University of Antwerp, Wilrijk, Belgium; 2 University Forensic Centre, University Hospital Antwerp (UZA), Edegem, Belgium; 3 Donders Institute for Brain, Cognition and Behaviour, Radboud University, Nijmegen, The Netherlands; 4 Forensic Psychiatric Centre Pompestichting, Nijmegen, The Netherlands; 5 Centre for Advances in Behavioural Science, Faculty of Health and Life Sciences, Coventry University, Coventry, United Kingdom; Centre for Criminology (Kriminologische Zentralstelle – KrimZ), Wiesbaden, Germany

**Keywords:** child sexual offending, self-regulation problems, neuropsychology of child sexual offending behavior, reinforcement learning

## Abstract

Self-regulation problems are critically involved in the onset and the maintenance of sexual offending behavior against children. Studying the neuropsychological underpinnings of these problems could help deepen our understanding of this contributing factor and, thus, of sexual offending behavior. Whereas most studies have examined executive functioning in relation to self-regulation problems in individuals convicted of sexual offenses against children (ISOCs), this review aimed to provide an overview of what is known about another process that is involved in self-regulation, that is reinforcement learning. The results of this review suggested that ISOCs are impaired in their ability to acquire and reverse stimulus-reward and stimulus-punishment associations relative to nonoffender controls, but similar to a control group of individuals convicted of nonsexual violent offenses. These reinforcement learning impairments were found to be more pronounced in nonpedophilic ISOCs than in pedophilic ISOCs. By paving the way towards a deeper understanding of the self-regulation problems seen in ISOCs, this review can help guide treatment strategies for ISOCs.

## Abstract (plain language summary)

**Background:**

Individuals convicted of sexual offenses against children (ISOCs) have difficulties to regulate their impulses and behaviors (i.e., self-regulation problems). These self-regulation problems have been shown to play a crucial role in the onset and the maintenance of their offending behavior, and should thus be targeted in the treatment of ISOCs.

**Why was this study done?:**

A review was conducted to enhance our understanding of the origins of the self-regulation problems among ISOCs. To this end, this review provided an overview of what is known about reinforcement learning in ISOCs, with this process being critically involved in self-regulation. Reinforcement learning is the process by which individuals learn to associate their actions with the outcomes that follow, and modulate their behavior in the service of these outcomes. In general, individuals will select behaviors that are associated with positive outcomes (i.e., rewards) and suppress behaviors that are associated with negative outcomes (i.e., punishments).

**What did the researchers find?:**

The results of this review suggested that ISOCs, especially ISOCs who were not diagnosed with a pedophilic disorder, are impaired in their ability to modulate their behavior on the basis of reward and punishment feedback relative to nonoffender controls, but similar to a control group of individuals convicted of nonsexual violent offenses.

**What do these findings mean?:**

The findings indicate that reinforcement learning impairments might underlie the self-regulation problems that are seen in ISOCs. Targeting the reinforcement learning impairments in treatment programs for ISOCs could therefore be necessary to tackle their self-regulation problems effectively and reduce the risk of further sexual offending.

## Highlights

- Individuals convicted of sexual offenses against children have difficulties to modulate their behavior on the basis of reward and punishment feedback, relative to nonoffenders.
- This reinforcement learning impairment is more pronounced in nonpedophilic than in pedophilic individuals convicted of sexual offenses against children.
- This impairment may underlie the self-regulation problems that are seen in individuals convicted of sexual offenses against children.
- Insight into the neuropsychological underpinnings of the self-regulation problems of individuals convicted of sexual offenses against children can help improve treatment strategies for sexual offending behavior against children.

Because of its high prevalence and devastating effects on the mental and physical health of victims (Barth et al., 2013; Maniglio, 2009; Pereda et al., 2009), sexual offending behavior against children is recognized by the World Health Organization as a priority health problem (World Health Organization, 2017). Societies around the world are, therefore, challenged to develop an adequate response to such behavior. Since the formulation of the Risk-Need-Responsivity (RNR) Model (Andrews et al., 1990; Bonta & Andrews, 2016), the idea that individuals convicted of sexual offenses against children (ISOCs) can be treated with psychological interventions has gained acceptance in most countries. This has led to the development and the implementation of therapeutic programs that are designed to reduce the risk of sexual recidivism in ISOCs. Two recent meta-analyses on sexual offender treatment effectiveness found that treatment reduces the risk of sexual reoffending by 33% and 26%, respectively (Gannon et al., 2019; Schmucker & Lösel, 2017). These findings highlight that some individuals convicted of sexual offenses, including ISOCs, respond well to treatment. However, the results from these meta-analyses also indicate that there is no truly effective intervention for many of these individuals, which necessitates the further improvement of existing treatment programs. To further develop treatment programs for ISOCs and improve therapeutic effectiveness, a thorough understanding of the factors that contribute to sexual offending behavior is essential. Furthermore, since ISOCs are a heterogeneous population (Robertiello & Terry, 2007), a sound understanding of the individual differences among ISOCs is also of importance. If we, for example, distinguish between pedophilic and nonpedophilic ISOCs (Groth et al., 1982; Seto, 2008), differences are found not only with respect to clinical features and underlying motivations to offend, but also in the criminogenic factors that contribute to their offending behavior (e.g., Eastvold et al., 2011; Strassberg et al., 2012; Suchy et al., 2009). Understanding the latter within-group differences will help to further develop treatment programs for ISOCs.

Self-regulation problems are identified as a key contributing factor to the onset and the maintenance of sexual offending behavior against children (Hanson et al., 2007; Hanson & Morton-Bourgon, 2005; Mann et al., 2010; Ward et al., 2006; Ward & Beech, 2017). In the meta-analysis by Hanson and Morton-Bourgon (2005), this factor emerged as one of the strongest predictors of sexual recidivism along with other indicators of “antisocial orientation” and indicators of “sexual deviancy”. In addition to predicting sexual recidivism, self-regulation problems were also found to predict nonsexual violent and general recidivism in individuals convicted of sexual offenses (Etzler et al., 2020; Hanson & Morton-Bourgon, 2005). This finding accords with the notion that self-regulation problems and the overlapping concept of “low self-control” constitute a major predictor of criminal behavior in general (Gottfredson & Hirschi, 1990).

Self-regulation problems also feature prominently in etiological theories of sexual offending, that describe self-regulation problems and low self-control as important etiological factors to the development of sexual offending behavior against children (e.g., Finkelhor, 1984; Seto, 2019; Ward et al., 1998). For example, the motivation-facilitation model of sexual offending against children argues that a sexual offense against a child will only occur when the desire to sexually abuse a child is not inhibited due to poor self-control (Seto, 2019). In accordance with the fact that self-regulation problems are a key factor in the onset and the maintenance of child sexual offending, improving self-regulation is a very important goal in the treatment of ISOCs (Fortune & Ward, 2017; Marshall & Marshall, 2017; Yates, 2013). The specific nature of the self-regulation problems is, however, not fully understood, which makes it difficult to select appropriate treatment strategies to remediate these problems.

In order to elucidate the nature of the self-regulation problems that are evident in ISOCs, it would be helpful to unravel the processes that underpin these problems. In the motivation-facilitation model of sexual offending against children, neuropsychological deficits in executive function are considered as a possible cause of self-regulation problems (Seto, 2019). By linking poor self-regulation to underlying neuropsychological deficits, the motivation-facilitation model of sexual offending against children is in harmony with the recent trend in psychiatry and psychology to integrate insights from neuroscience, and link psychopathology to disturbances in underlying neuropsychological mechanisms (e.g., Brazil et al., 2018; Insel & Cuthbert, 2015; Montague et al., 2012; Stephan & Mathys, 2014). As such an approach promises to provide new ways to understand important risk factors to sexual offending including self-regulation problems, furthering our understanding of the neuropsychological underpinnings of the self-regulation problems may help pave the way towards more effective treatment of this key contributing factor.

Up to now, most attention has been given to executive functions when studying the neuropsychological underpinnings of the self-regulation problems. Two recent systematic reviews showed that ISOCs are impaired in their executive functioning (Dillien et al., 2020b; Turner & Rettenberger, 2020). In these studies, executive function is considered an umbrella term that comprises a set of cognitive processes that enable individuals to regulate and control their actions and engage in purposeful, future-oriented behavior (e.g., Kelley et al., 2015; Salehinejad et al., 2021; Wagner & Heatherton, 2016). Core executive functions include: The ability to suppress a prepotent but inappropriate response to a stimulus to select a more appropriate one (i.e., inhibitory control), the ability to adjust one’s behavior in response to changing situational demands (i.e., cognitive flexibility), and the ability to simultaneously store and manipulate information in one’s mind (i.e., working memory) (Diamond, 2013). These processes are purely cognitive (i.e., emotion-independent) and therefore sometimes referred to as “cold” cognitive processes (Salehinejad et al., 2021). As described in the review by Dillien et al. (2020b), ISOCs are heterogeneous in their executive dysfunctions, with pedophilic and nonpedophilic ISOCs showing distinct executive function profiles. Whereas both the pedophilic and the nonpedophilic ISOCs demonstrated impaired inhibitory control, the nonpedophilic ISOCs additionally showed impairments in cognitive flexibility and impairments in visuospatial working memory relative to nonoffender controls (see Dillien et al., 2020b).

Notwithstanding the role these executive dysfunctions play in the self-regulation problems that are seen in ISOCs, the emphasis on these dysfunctions in the sexual offender literature has come at the expense of attention to other cognitive processes that also underlie self-regulation, including affective decision-making, facial affect recognition, and reinforcement learning. These cognitive processes involve cognitive abilities that relate to emotion, reward, motivation, and social evaluation, and are therefore sometimes referred to as “hot” cognitive processes or emotion and motivation-related cognitive processes (e.g., De Brito et al., 2013; Jonker et al., 2015; Kelley et al., 2019; Kelley et al., 2015; Salehinejad et al., 2021; Schuck et al., 2018; Wagner & Heatherton, 2016).

One of these emotion and motivation-related cognitive processes, that is reinforcement learning, is critically involved in the inhibition of unwanted behavior. Because of this, this process attracted considerable attention in the offender literature, where reinforcement learning impairments have been described in violent populations, particularly in those with antisocial and/or psychopathic personality traits (e.g., Budhani et al., 2006; De Brito et al., 2013). *Reinforcement learning* is the process by which individuals learn to associate their actions with the outcomes that follow, and modulate their behavior in the service of these outcomes, which function as reinforcement. In general, individuals will select behaviors that are associated with high-valued outcomes (i.e., rewards) and suppress behaviors that are associated with negative outcomes (i.e., punishments). In addition to acquiring contingencies (i.e., acquisition learning), individuals must also be able to adapt their behavior when contingencies change and previously rewarded behaviors are no longer appropriate (i.e., reversal learning) (Behrens et al., 2008; Rushworth et al., 2012).

As reinforcement learning impairments interfere with the ability to suppress behavioral tendencies that are associated with punishment, these impairments will increase the likelihood that antisocial tendencies are acted on, instead of being regulated or controlled. This notion probably explains the link between reinforcement learning impairments and antisocial behavior. In line with this notion, reinforcement learning impairments could also play a role in the self-regulation problems that are seen in ISOCs. Taking a closer look at reinforcement learning in ISOCs might therefore help deepen our understanding of the self-regulation problems of ISOCs and ultimately of sexual offending behavior. This review presents a summary of our own work and that of others on reinforcement learning in ISOCs. This overview of what is currently known about the presence of reinforcement learning impairments in ISOCs can serve as a basis for further research on this topic.

## Method

The reviews by Dillien et al. (2020b) and Turner and Rettenberger (2020) were used as a starting point for the current review. Studies that were included in these reviews, as well as the reference lists of the included studies, were examined to identify studies that provided data on reinforcement learning in ISOCs. Additionally, the Web of Science was searched for publications on reinforcement learning in ISOCs that had appeared since these reviews.

To be included in this review, studies had to measure reinforcement learning by means of behavioral tasks such as probabilistic reversal learning tasks (e.g., Budhani et al., 2006), passive avoidance tasks (e.g., Blair et al., 2004), or more experimental reinforcement learning tasks. Studies that provided data on choice tasks with risky outcome such as the Game of Dice Task (GDT; Brand et al., 2005) or the Iowa Gambling Task (IGT; Bechara et al., 2000) were also considered. Although these latter tasks are not “pure” measures of reinforcement learning, performance on these tasks also requires the ability to use reinforcement to modulate one’s behavior and therefore also tap into reinforcement learning. Further inclusion criteria for the studies were as follows: 1) studies had to be peer-reviewed, 2) studies had to be published in English, and 3) studies had to compare the performance of adult ISOCs to that of nonoffender samples or other offender populations.

In total, five articles were included in this review. The characteristics of the included studies are shown in Table 1.

**Table 1:** The Characteristics of the Included Studies

Author	Title	Participants	Processes measured & measures used
Leue et al. (2008)	Reinforcement sensitivity of sex offenders and non-offenders: An experimental and psychometric study of reinforcement sensitivity theory	50 paraphilic individuals convicted of sexual offenses vs. 48 nonparaphilic individuals convicted of sexual offenses vs. 51 nonoffender controls	Reversal learning, measured by a choice task
Dillien et al. (2019)	Reinforcement learning in child molesters	59 ISOCs vs. 33 individuals convicted of nonsexual violent offenses vs. 36 nonoffenders controls Supplementary analysis: 17 pedophilic ISOCs vs. 30 nonpedophilic ISOCs	Reinforcement learning, measured by a probabilistic reversal learning task
Dillien et al. (2020a)	Impairment of both reward and punishment learning in males who have sexually offended against a child	57 ISOCs vs. 31 individuals convicted of nonsexual violent offenses vs. 33 nonoffender controls Supplementary analysis: 21 pedophilic ISOCs vs. 36 nonpedophilic ISOCs	The ability to learn from negative outcomes (i.e., punishment learning) and the ability to learn from positive outcomes (i.e., reward learning), measured by a passive avoidance task
Turner et al. (2018)	Response inhibition and impulsive decision-making in sexual offenders against children	63 ISOCs vs. 63 nonoffender controls	Affective decision-making, measured by the Iowa Gambling Task and the Game of Dice Task
Turner et al. (2020)	Deviant sexual interests but not antisocial behaviors are associated with deficits in executive functioning in individuals convicted of sexual offenses against children	70 ISOCs vs. 49 individuals convicted of sexual offenses against adults vs. 54 individuals convicted of nonsexual offenses vs. 70 nonoffender controls	Affective decision-making, measured by the Iowa Gambling Task

*Note.* ISOCs = individuals convicted of sexual offenses against children.

## Results

Three of the five included studies evaluated the performance of ISOCs on reinforcement learning tasks (Dillien et al., 2019, 2020a; Leue et al., 2008). The two other studies used choice tasks with risky outcome (Turner et al., 2018, 2020). Since these latter tasks are not “pure” measures of reinforcement learning, the results from these studies are presented as a complement to the studies that employed reinforcement learning tasks.

### Studies That Used Reinforcement Learning Tasks

Leue et al. (2008) examined reinforcement learning in a mixed group of individuals convicted of sexual offenses, including ISOCs. These individuals convicted of sexual offenses were divided in 50 paraphilic individuals and 48 nonparaphilic individuals, and performances of these groups were compared to the performance of 51 nonoffender controls. In the task used (Avila & Parcet, 2000), participants are instructed to earn as many points as possible by pushing one of two buttons. Whereas both buttons are rewarded in the first phase of the task, contingencies change after 200 trials and one of the buttons no longer produces reward. Successful choice behavior after this change in contingencies requires intact reversal learning to allow participants to flexibly adapt to these changes. Results from this study showed that nonparaphilic individuals convicted of sexual offenses were less able to adapt their behavior in response to changes in stimulus-reward contingencies, relative to the nonoffender controls and the paraphilic individuals convicted of sexual offenses.

Dillien et al. (2019) were the first to focus on reinforcement learning in a group that consisted solely of ISOCs. This study, moreover, allowed to specify the nature of the hypothesized reinforcement learning impairments by using a probabilistic reversal learning task that tested for both acquisition learning and reversal learning (Budhani et al., 2006). This computerized task instructed participants to earn as much fake money as possible by choosing the correct animal in six animal pairs that were presented throughout the task. In the first phase of the task (i.e., the acquisition phase) participants were required to learn to choose the correct animal. Feedback was presented immediately after choosing one of the animals in the form of fake money given (i.e., reward) or withdrawn (i.e., punishment). Four animal pairs had a 100-0 contingency: choosing the correct animal was always rewarded and choosing the incorrect animal was always punished. The other two animal pairs had an 80-20 contingency (i.e., a probabilistic reinforcement contingency): choosing the correct animal was rewarded in 80% of the trials but punished in 20% of the trials, choosing the incorrect animal was punished in 80% of the trials but rewarded in 20% of the trials. For two of the six animal pairs, the reward-punishment contingencies reversed after 40 trials (i.e., reversal phase), which required participants to reverse previously learned associations and adapt their choice behavior. The contingencies of the other pairs remained constant throughout the task. The results of this study showed that ISOCs are impaired in their ability to acquire stimulus-reinforcement contingencies and to adjust their (choice) behavior in response to contingency changes. This general reinforcement learning impairment that affects both acquisition learning and reversal learning was found relative to nonoffender controls. The control group of individuals convicted of nonsexual violent offenses did not differ significantly from ISOCs or from nonoffender controls. This study, furthermore, showed that the nonpedophilic ISOCs were more severely impaired in their reinforcement learning abilities than the pedophilic ISOCs.

In another study, with an overlapping sample, Dillien et al. (2020a) further specified the acquisition learning impairments seen in ISOCs by using a passive avoidance task (Blair et al., 2004). This task allowed to differentiate between the ability to learn from negative outcomes or punishment (i.e., punishment learning) versus positive outcomes or reward (i.e., reward learning). In the passive avoidance task, participants are presented with eight different stimuli, of which four are associated with reward (i.e., participants earn points when they press spacebar when these stimuli are showing) and four are associated with punishment (i.e., participants lose points when they press spacebar when these stimuli are showing). Participants are instructed to earn as many points as possible by responding to the stimuli that are associated with reward and withhold from responding to the stimuli that are associated with punishment. After responding or not responding, feedback is given regarding how many points were won or lost. The individual stimuli are associated with different reinforcement values (+1, +50, +100, and +200 points for the stimuli that are associated with reward; -1, -50, -100, and -200 points for the stimuli that are associated with punishment). The results of this study showed that both the ability to acquire stimulus-reward contingencies and the ability to acquire stimulus-punishment contingencies are compromised in ISOCs relative to nonoffender controls. No differences were found between ISOCs and the control group of individuals convicted of nonsexual violent offenses, who also differed from nonoffender controls with respect to the ability to acquire stimulus-punishment contingencies. The impairment in the ability to acquire stimulus-punishment contingencies therefore seems to be shared among individuals convicted of violent offenses. This impairment was, moreover, found to be the most severe in nonpedophilic ISOCs, with the pedophilic ISOCs not differing significantly from the nonoffender controls.

### Studies That Employed Choice Tasks With Risky Outcome

Turner et al. (2018) assessed affective decision-making in a sample of 63 ISOCs and 63 nonoffender controls using the IGT (Bechara et al., 2000) and the GDT (Brand et al., 2005). In the IGT, participants are instructed to earn as much fake money as possible by selecting cards from four decks of cards with different reinforcement contingencies. Decks A and B are associated with high wins but also with large losses. Decks C and D are associated with smaller wins but also with smaller losses, with these decks resulting in a greater gain in the long run. On the basis of feedback regarding how much money was lost or won after a card selection, participants learn to avoid the disadvantageous decks (i.e., decks A and B) and select cards from the advantageous decks (i.e., decks C and D). In this study, images of nude adults and nude children that were taken from the Not Real People picture set (Pacific Psychological Assessment Corporation, 2004) were used as the backside of the cards. This modification was done to test for the possibility that emotional or sexual arousing stimuli may trigger or worsen decision-making impairments. Similar to the IGT, the GDT instructs participants to earn as much fake money as possible. In this task, participants have to guess which number will be thrown by a virtual dice. When participants guess the correct number, they win money. When participants, however, guess the incorrect number, they lose money. Participants can either bet on a single number, or on two, three, or four numbers. Betting on one or two numbers are high-risk choices as they lead to potential high gains but also to potential high losses. In contrast, when betting on three or four numbers, participants make low-risk choices that are associated with lower wins but also lower losses. The results on the GDT showed that the ISOCs chose the high-risk options more frequently than the nonoffender controls with the latter group selecting the low-risk options more frequently. ISOCs were also found to be less inclined to use negative feedback to shift to more low-risk options in the following trial, relative to nonoffender controls. On the IGT, ISOCs scored worse than the nonoffender controls with this difference approaching significance (*p* = .05). This finding suggests that ISOCs are less able than nonoffender control to learn to select more advantageous cards on the basis of reward and punishment feedback.

In contrast to the latter finding, a very recent study by the same research group (Turner et al., 2020) failed to find differences between 70 ISOCs, 49 individuals convicted of sexual offenses against adults, 54 individuals convicted of nonsexual offenses, and 70 nonoffender controls on the IGT.

## Discussion

An impaired ability to regulate oneself has been described as an important factor in the onset of sexual offending behavior against children (e.g., Finkelhor, 1984; Seto, 2019; Ward et al., 1998). It has, moreover, been identified as a factor that contributes strongly to sexual recidivism (e.g., Hanson et al., 2007; Hanson & Morton-Bourgon, 2005; Mann et al., 2010), and thus as an important treatment target. The current review sought to further our understanding of the neuropsychological underpinnings of this contributing factor by summarizing what is known about reinforcement learning in ISOCs. The few studies that have focused on this topic indicated that ISOCs are impaired in their ability to acquire and reverse stimulus-reward and stimulus-punishment associations relative to nonoffender controls. ISOCs seem to share their impaired ability to acquire stimulus-punishment contingencies with individuals convicted of nonsexual violent offenses who scored similarly to ISOCs and significantly different from nonoffender controls with respect to this ability. The study by Turner et al. (2018) that evaluated the performances of ISOCs on choice tasks with risky outcome is in line with these conclusions by showing that ISOCs have difficulties in using reward and punishment feedback to modulate their behavior, relative to nonoffender controls (but see Turner et al., 2020).

When discussing these results, it is important to realize that the findings of reinforcement learning impairments are on a group level, and individual differences in performance were rather large. This implies that a portion of the ISOCs will present with reinforcement learning deficits that affect self-regulation, while other ISOCs will show intact reinforcement learning. The studies that were presented in this review suggested that nonpedophilic ISOCs show more pronounced reinforcement learning impairments than pedophilic ISOCs (Dillien et al., 2019, 2020a; Leue et al., 2008). A similar pattern was found in studies on executive dysfunctions, which were also found to be more marked in nonpedophilic ISOCs relative to pedophilic ISOCs (see Dillien et al., 2020b).

The finding that pedophilic ISOCs are less impaired than nonpedophilic ISOCs in their executive functioning and in their reinforcement learning capacity could mean three things. First, it is possible that pedophilic ISOCs as a group show less self-regulation problems than nonpedophilic ISOCs. Some indirect evidence for the assumption that pedophilic ISOCs show intact self-regulation capabilities can be found in studies on ISOCs who have offended against children during their professional (e.g., a schoolteacher) or voluntary (e.g., sport coach) work (Turner et al., 2014). These ISOCs constitute a subgroup that is characterized by high rates of pedophilia. This subgroup is found to be less impulsive, antisocial, and psychopathic relative to other ISOCs. Furthermore, the fact that these individuals were able to maintain a position in which they were entrusted with the care of children, as well as the fact that they were able to keep their victims from disclosing the abuse for some time demonstrates the presence of some degree of self-regulation (Turner et al., 2014; Turner & Rettenberger, 2020). The latter fits with the idea that pedophilic ISOCs generally offend in a planned and deliberate manner and use grooming strategies as a way to establish trusting relationships with potential victims and their parents (see also Eastvold et al., 2011; Suchy et al., 2009). Although this evidence suggests that pedophilic ISOCs have intact self-regulation capabilities, it is indirect and requires confirmation from further studies. A second possibility is that pedophilic ISOCs do show self-regulation problems that, however, do not originate from neuropsychological impairments, but are situationally activated. As stated in the motivation-facilitation model of sexual offending (Seto, 2019), transient factors such as being intoxicated, experiencing extreme stress or negative affect can temporarily deplete self-regulation capabilities. A further possibility is that whereas nonpedophilic ISOCs show general self-regulation problems, pedophilic ISOCs exhibit self-regulation problems specifically in a sexual context. The results of the study by Turner et al. (2018), who studied the affective decision-making ability of ISOCs in the presence of salient sexual stimuli, are in line with this possibility. More specifically, this study found that those ISOCs who showed intense pedophilic sexual interests were most affected in their affective decision-making ability by the presence of sexual stimuli.

Notwithstanding this, this review indicated that the neuropsychological impairments that are involved in the self-regulation difficulties of ISOCs are not limited to purely cognitive executive dysfunctions, but also include dysfunctions in the cognitive processes that are related to emotion, reward, motivation, and social evaluation, particularly reinforcement learning dysfunctions. Since the control group of individuals convicted of nonsexual violent offenses also demonstrated an impaired ability to acquire stimulus-punishment associations in the study by Dillien et al. (2020a), this review furthermore suggested that some of the reinforcement learning impairments seen in ISOCs are also present in individuals convicted of nonsexual violent offenses. Previous studies that investigated reinforcement learning in individuals with violent offenses and a diagnosis of psychopathy and individuals with violent offenses and a diagnosis of antisocial personality disorder (e.g., Budhani et al., 2006; De Brito et al., 2013) have come to similar conclusions. Taken together, this review indicated that both ISOCs and individuals convicted of nonsexual violent offenses have impairments in their reinforcement learning abilities that probably have consequences for their ability to regulate their behavior. As noted, this especially holds for nonpedophilic ISOCs, and not so much for pedophilic ISOCs. In pedophilic ISOCs, other mechanisms could lead to self-regulatory failure, but it is also possible that the self-regulation capabilities of pedophilic ISOCs are largely intact and other facilitators besides self-regulation problems are involved in their sexual offending behavior (Seto, 2019).

Whereas purely cognitive executive dysfunctions reflect a disruption of dorsolateral and ventrolateral prefrontal cortical function, dysfunctional emotion and motivated-related cognitive processes in general and reinforcement learning impairments in particular involve dysfunction of ventromedial pathways that connect the mesolimbic reward structures (e.g., the amygdala and the insula) to ventromedial prefrontal and orbitofrontal cortical areas of the brain (Jonker et al., 2015; Kelley et al., 2019; Kelley et al., 2015; Salehinejad et al., 2021; Schuck et al., 2018; Wagner & Heatherton, 2016). Several magnetic resonance imaging (MRI) studies indeed reported structural and functional abnormalities in limbic and prefrontal structures including the amygdala and the orbitofrontal cortex in ISOCs (Jordan et al., 2020; Kirk-Provencher et al., 2020; Mohnke et al., 2014). Two recent functional imaging studies, moreover, revealed diminished resting state functional connectivity between limbic structures (especially the amygdala) and prefrontal cortical areas (including the dorsolateral prefrontal cortex and the orbitofrontal cortex extending to the anterior cingulate cortex) in nonpedophilic ISOCs relative to nonoffender controls (Kärgel et al., 2015) and in pedophilic ISOCs relative to nonoffending individuals with pedophilic preferences and nonoffender controls (Kneer et al., 2019). As suggested by the authors, the finding of reduced fronto-limbic functional connectivity may constitute a neural correlate of the self-regulation impairments that are seen in ISOCs and may be part of the neurobiological mechanisms underlying child sexual offending behavior (Kärgel et al., 2015; Kneer et al., 2019). In line with the finding of some reinforcement learning impairments in the control group of individuals convicted of nonsexual violent offenses, fronto-limbic abnormalities have also been reported in populations that consist of individuals convicted of nonsexual violent offenses (e.g., individuals with violent offenses and a diagnosis of psychopathy and individuals with violent offenses and a diagnosis of antisocial personality disorder: Motzkin et al., 2011; Völlm et al., 2004). This indicates that such abnormalities should probably be seen as neurobiological correlates of violent offending behavior in general rather than as being specific to sexual offending behavior (Kirk-Provencher et al., 2020).

These findings have clinical implications. For those ISOCs whose self-regulation problems are grounded in neuropsychological impairments, remediating these neuropsychological impairments in treatment programs for ISOCs (i.e., cognitive remediation interventions) could be necessary to tackle their self-regulation problems effectively. Cognitive remediation interventions should be deficit-specific (Baskin-Sommers et al., 2015). Therefore, these interventions should not only target the executive dysfunctions that are seen in ISOCs, but should also address the reinforcement learning deficits of ISOCs to be most successful. Although cognitive remediation interventions have not been widely applied to offender populations, there are some studies on the effects of such interventions with offenders showing promising results, in particular in reducing neuropsychological deficits and antisocial behavior (Baskin-Sommers et al., 2015; Brunton & Hartley, 2013; Shumlich et al., 2019). When ISOCs suffer from sexual preoccupation and/or paraphilic disorders, these interventions might need to be combined with testosterone-lowering medications. As found in the study by Sauter et al. (2021), individuals convicted of sexual offenses who are diagnosed with paraphilic disorders and who suffer from deficient self-regulation, showed improved general and sexual self-regulation after the initiation of hormonal treatment. This finding suggests that testosterone-lowering medications play an important role in the treatment of the self-regulation problems of individuals convicted of sexual offenses with severe paraphilic disorders, probably because intense sexual urges and/or fantasies would otherwise hinder them from benefiting from self-regulation enhancing interventions.

Although this review offers an insight into the reinforcement learning ability of ISOCs, it should be taken into account that a very limited body of evidence was available on reinforcement learning in ISOCs. Moreover, some of the included studies had methodological shortcomings that might have biased their results. For example, Leue et al. (2008) included ISOCs and individuals convicted of sexual offenses against adults in their sample, even though these groups are found to differ in their (socio-) cognitive functioning (Gudjonsson & Sigurdsson, 2000; Joyal et al., 2014). Likewise, the studies by Dillien et al. (2019, 2020a) were unable to untangle the influence of age and IQ on the reinforcement learning impairments that were seen in ISOCs. Therefore, the conclusions of this review need to be considered with caution and need to be corroborated by future research that overcomes the methodological shortcomings of the included studies. This highlights the need to perform further studies on reinforcement learning in ISOCs.

### Conclusion

This review indicates that ISOCs have difficulties to modulate their behavior on the basis of reward and punishment feedback, which may underlie their self-regulation problems. By deepening our understanding of the neuropsychological underpinnings of the self-regulation problems in ISOCs, this review can help improve current treatment strategies for ISOCs and reduce the risk of further sexual offending.
